# Influence of protein fold stability on immunogenicity and its implications for vaccine design

**DOI:** 10.1080/14760584.2017.1306441

**Published:** 2017-03-24

**Authors:** Sandra Scheiblhofer, Josef Laimer, Yoan Machado, Richard Weiss, Josef Thalhamer

**Affiliations:** ^a^ Department of Molecular Biology, University of Salzburg, Salzburg, Austria

**Keywords:** Protein stability, fold stability, conformational stability, stabilization, destabilization, protein variants, immunogenicity, processing, recombinant vaccines

## Abstract

**Introduction**: In modern vaccinology and immunotherapy, recombinant proteins more and more replace whole organisms to induce protective or curative immune responses. Structural stability of proteins is of crucial importance for efficient presentation of antigenic peptides on MHC, which plays a decisive role for triggering strong immune reactions.

**Areas covered**: In this review, we discuss structural stability as a key factor for modulating the potency of recombinant vaccines and its importance for antigen proteolysis, presentation, and stimulation of B and T cells. Moreover, the impact of fold stability on downstream events determining the differentiation of T cells into effector cells is reviewed. We summarize studies investigating the impact of protein fold stability on the outcome of the immune response and provide an overview on computational methods to estimate the effects of point mutations on protein stability.

**Expert commentary**: Based on this information, the rational design of up-to-date vaccines is discussed. A model for predicting immunogenicity of proteins based on their conformational stability at different pH values is proposed.

## 1. Introduction

While traditional vaccines mostly employ live-attenuated or dead pathogens, with the advent of recombinant protein manufacturing, modern vaccines only incorporate defined, immunologically relevant components thereof. However, recombinant proteins such as the hepatitis B surface antigen often display limited immunogenicity in the absence of pathogen-associated molecular patterns (PAMPs) that are present in traditional vaccines. Thus, a lot of effort has been directed at the development of effective yet safe adjuvants that can mimic the immunostimulatory capacity of full pathogens [1]. One aspect in vaccine design that has only recently come into focus is the recombinant protein itself and especially its structural stability. In this review, we will discuss how conformational stability affects antigen processing and presentation and thus immunogenicity and immune polarization.

To be able to eliminate virally infected or tumor cells and to support antibody production by B cells, T cells first have to interact with cells displaying information about antigens they have synthesized or taken up. Peptides derived from endogenously produced antigens are presented to CD8+ T cells via major histocompatibility complex (MHC) class I molecules, leading to their differentiation into cytotoxic T cells. In contrast, specialized antigen-presenting cells (APCs) such as dendritic cells (DCs), B cells, and macrophages present peptides from ingested antigens in the context of MHC class II molecules to CD4+ T cells, inducing their activation and differentiation into T helper subsets [2].

### 1.1. Presentation of the ‘inner world’ on MHC class I molecules

In healthy cells, MHC I molecules present peptides from autologous proteins (against which CD8+ cells have been tolerized); however, upon viral infection or cancer development also, foreign antigenic peptides are displayed, enabling CD8+ T cells to identify and destroy infected or tumor cells. Processing of intracellular proteins starts with cleavage of normal as well as aberrant molecules by the proteasome. Unfolding of proteins is a critical upstream process before cleavage into peptides can take place [3]. Subsequently, some of these peptides enter the endoplasmic reticulum (ER) via transporter associated with antigen processing (TAP) [4]. Together with empty MHC I molecules and the chaperones tapasin, calreticulin, and protein disulfide isomerase ERp57 [5], TAP forms a peptide-loading complex and transports peptides into the lumen of the ER. At this state, many peptides are ‘tested’ for binding and only those with a low off-rate, a length of 8–10 amino acids, and amino acid side chains (so-called ‘anchor residues’) complementary to the peptide-binding groove of the MHC I molecule are presented on the cell surface after leaving the ER [6,7].

### 1.2. Presentation of the ‘outer world’ on MHC class II molecules

APCs employ several mechanisms to capture antigens. Receptor-mediated endocytosis via clathrin-coated vesicles represents a specific uptake process as antigen is bound by specific cell surface receptors on APCs [8]. Macropinocytosis, on the other hand, is an unspecific, actin-dependent mechanism of uptake of soluble extracellular material [9]. DCs and macrophages preferentially take up foreign antigens via phagocytosis [10]. This endocytic, actin-dependent uptake of particulate antigen (>3 µm, e.g. opsonized pathogens or apoptotic cells) is often receptor mediated, but can also be an unspecific process. The internalized antigens are collected in early endosomes, macropinosomes, or phagosomes, which subsequently fuse with late endosomal–lysosomal vesicles providing the proper prerequisites, i.e. low pH and presence of proteases, for antigen processing and MHC II peptide loading [11]. As with proteasomal processing, (partial) unfolding of proteins is a prerequisite for efficient degradation by endosomal–lysosomal proteases.

MHC II molecules are assembled in the ER and associate with the invariant chain Ii, which fills the peptide-binding groove. The Ii–MHC II complexes traffic through the Golgi apparatus and reach the plasma membrane, where they become internalized via clathrin-mediated endocytosis [12]. Subsequently, the complexes travel from early endosomes to the endosomal–lysosomal antigen-processing compartment. Here, resident proteases generate antigenic peptides and degrade the invariant chain, resulting in a fragment designated class II–associated invariant chain peptide (CLIP), which remains bound to the peptide-binding groove [13]. Upon removal of CLIP by the MHC-II-like chaperone human leukocyte antigen DM (HLA-DM) (H2-M in mice) [14], antigenic peptides with higher binding affinity can take its place and MHC II molecules reach the plasma membrane with the help of endosomal–lysosomal tubules [15]. This classical model has been complemented by the ‘bind first, cut/trim later’ hypothesis, which proposes that MHC molecules can scan the length of a protein for epitopes with high binding affinity, which are then cut and trimmed outside of the MHC-binding groove [16]. Recent data indicate that both models are valid and that the nature of the antigen determines which type of processing is employed. Immunodominant epitopes that are susceptible to cathepsin cleavage have been shown to follow the ‘bind first, cut/trim later’ model, while cathepsin-resistant epitopes were processed in the classical way. Interestingly, the latter were found in pathogen-derived antigens, while autoantigen-derived epitopes showed high cathepsin resistance [17].

### 1.3. Presenting exogenous antigens on MHC class I molecules – cross-presentation

Naive CD8+ T cells have to become activated by professional APCs to develop into effector cytotoxic T lymphocytes (CTLs) capable of eliminating transformed or infected cells. In case the APCs themselves are not infected, they have to capture foreign antigens and present them on MHC I molecules. This mechanism, designated cross-presentation, *in vivo* is mainly employed by specific DC subsets, which have adapted their endocytic and phagocytic pathways accordingly, as extensively reviewed elsewhere [18]. In vaccine development, the use of long peptides or full-length proteins requires cross-presentation for induction of protective CD8+ immune responses [19]. Proteins that are not efficiently processed in the antigen-processing compartment may enter the cross-presentation pathway as discussed later in this review.

## 2. Qualitative and quantitative aspects of CD4+ T-cell polarization

Interaction of CD4+ T cells via their T-cell receptors (TCR) with peptide-MHC (pMHC) II complexes leads to activation and differentiation into effector subsets such as TH1, TH2, TH17, TH22, inducible regulatory T cells (iTreg), or follicular T helper cells (TfH) [20,21]. The cytokine milieu established by (mainly myeloid) APCs and other tissue-resident cell types is decisive for the pathway of differentiation which is pursued. Upon pathogen encounter, APCs become activated by PAMPs, and they upregulate their MHC II and co-stimulatory molecule expression and also start production of cytokines. These cytokines direct differentiation of T helper subsets by binding to specific surface receptors, followed by activation of Janus kinase (JAK)/signal transducer and activator of transcription (STAT) pathways, and regulation of transcription factors at the switch points of cell linage [22].

Presence of interleukin (IL)-12 during T-cell activation promotes TH1 differentiation. TH1 cells produce the key effector cytokine IFN-γ and typically express transcription regulator T-bet. TH1 cells are crucial for cell-mediated defense against bacterial and viral infections, but also exert detrimental effects in the context of autoimmunity.

In contrast, TH2 induction seems to represent an endogenous or default pathway as no specific cytokine inevitable for TH2 differentiation *in vivo* could be identified. However, TH2 induction is promoted by IL-2, IL-4, IL-25, IL-33, and thymic stromal lymphopoietin (TSLP), leading to anti-(extracellular) parasitic reactions, but also to exaggerated responses towards harmless environmental antigens in the course of type I allergies. Characteristic for this T helper type is upregulation of transcription factor GATA binding protein 3 (GATA-3) and production of the cytokines IL-4, IL-5, and IL-13 [23].

Being involved in responses against extracellular bacterial and fungal infections [24], autoimmune diseases including encephalomyelitis [25], but also tumor immunology [26], TH17 cells have been described as expressors of transcription factor RORyt and producers of IL-17, IL-21, and/or IL-22 [27]. Their differentiation is induced by the presence of IL-6 and TGF-β and further amplified and stabilized by IL-21 and IL-23 [28].

Characterized by expression of aryl hydrocarbon receptor as key transcription factor and production of inflammatory cytokines IL-22 and TNF-α, TH22 cells play a substantial role in inflammatory and autoimmune disorders such as psoriasis, atopic dermatitis, rheumatoid arthritis, systemic lupus erythematosus, and type 1 and 2 diabetes [29].

In contrast to the T-cell subsets listed above, inducible Tregs, which differentiate in the presence of IL-2 and TGF-β and express transcription factor Foxp3, are responsible for the downregulation of exaggerated immune responses, limiting the degree of inflammation and induction of tolerance [30]. Several suppressive mechanisms have been described: (a) cell–cell contact-mediated suppression by interaction of inhibitory receptors such as CTLA-4 with CD80/86 and MHC molecules on DCs or delivery of granzyme B to effector T cells driving them into apoptosis; (b) metabolic disruption of effector T cells by delivery of cyclic adenosine monophosphate (cAMP) to effector T cells or via targeting of adenosine receptors on effector T cells or by deprivation of effector T cells through consumption of the growth factor IL-2; and (c) the secretion of inhibitory cytokines IL-10, IL-35, and TGF-β, which act on both T cells and DCs [31].

Until recently it was unclear whether TfH represent an independent T helper subset or originate from CD4^+^ T cells that have already determined their fates towards one of the other subsets. The development of TfH cells depends on expression of the transcriptional repressor Bcl-6, and they are characterized by production of CXCR5 and programmed cell death protein 1 (PD-1) [32]. Following their differentiation, TfH cells migrate to B-cell follicles within secondary lymphoid organs, where they are essential for germinal center formation and promote antibody class-switching and affinity maturation of B cells and establishment of B memory responses [20].

In the classical model of immune polarization, cytokines have been described as the key factors driving immune polarization. However, most of the studies dealing with mechanisms determining T helper cell differentiation have been performed without considering variations in antigen amount or concentration or the nature of the antigen itself. These aspects increasingly attract attention, and recent data point to an important role for TCR signal strength in directing CD4+ T-cell differentiation. Early *in vitro* studies demonstrated that strong TCR signaling preferentially induces TH1 polarization, whereas weak TCR signals promote the induction of TH2 cells [33]. As TCR signaling depends on the affinity of a pMHC II complex for a specific TCR as well as on the density of pMHC II complexes on the surface of an APC, peptides with different affinities and/or different doses of antigen determine the fate of naive CD4+ T cells receiving an initial TCR stimulus. However, adjustment of antigen dose to compensate for lower affinity (or vice versa) does not necessarily result in the same immunological outcome *in vivo*, pointing to the ability of the TCR to distinguish between antigenic quality and quantity [34,35]. It was found that whereas comparable proliferative responses can be obtained by application of either large doses of weak ligand peptides or low doses of strong ligand peptides, independent of dosage, application of high-affinity peptides leads to prolonged interaction between APC and CD4+ T cells, resulting in altered downstream gene expression in the latter [35]. Moreover, it was recently demonstrated by intravital imaging techniques that the strength of TCR signaling overrules the determination of polarization mediated by cytokines. At high antigen doses, DC activated by exposure to TH2-promoting adjuvants (papain and Schistosomal egg antigen) primed T cells towards a TH1 phenotype, whereas at low doses even TH1-polarizing adjuvants (lipopolysaccharide [LPS] and CpG oligodeoxynucleotides [CpG]) could not prevent differentiation into TH2 cells [36]. Depletion of CD4+ T cells with a high affinity for a certain pMHC II complex during a polyclonal response preferentially leads to TH2 differentiation [37].

For efficient generation of TfH cells, prolonged presentation of the respective antigenic peptides, as well as high affinity of TCR for pMHC II complexes, both resulting in strong and sustained TCR signaling, is required [38]. The presence of IL-6 and TGF-β abrogates the potential of naive CD4+ T cells to differentiate into TH1 or TH2 but promotes TH17 polarization at high antigen doses in combination with strong TCR signaling, whereas weak TCR signaling under a similar cytokine milieu favors Treg differentiation [39]. In summary, the length of interaction between TCR and pMHC II complexes and the strength of TCR signaling, together with activation of co-stimulatory molecules and cytokine-derived signals, shapes the differentiation program of T cells [22].

## 3. Impact of antigen stability on immunogenicity

Taking into account that conformational stability greatly influences the intracellular fate of antigens during processing in APCs (from their uptake to the final presentation of antigenic peptides), it is obvious that this protein-inherent property has great impact on immunogenicity and immune polarization. The term conformational or fold stability refers to the capacity of a protein to maintain its three dimensional (often biologically active) fold when subjected to physical or chemical stresses [40]. The nature of the stress, i.e. pH, denaturant, temperature, or redox environment, determines the unfolding pathway and the unfolded state of a protein [41]. For example, acidification during endolysosomal maturation may lead to protonation of groups, which in turn destabilize the protein by both destroying interactions between groups with opposite charge and increasing electrostatic repulsion with other protonated groups [42]. On the other hand, disulfide bonds can be reduced in the endolysosomal compartment by gamma interferon-inducible glutathione reductase (GILT), which leads to the unfolding of proteins stabilized by disulfide bonds [43]. Depending on their conformational stability, under these denaturing conditions, antigens sooner or later unfold, rendering the peptide backbone accessible for proteases. Hence, intrinsic stability of antigens greatly affects the kinetics of proteolytic degradation, thereby determining the availability of appropriate antigenic peptides for MHC II loading and, ultimately, immunogenicity and T-cell polarization.

Fold stability can also directly influence the availability of B-cell epitopes. Destabilization of a protein can lead to unfolding of the tertiary structure and thus loss of conformational epitopes. This is a common approach for designing hypoallergens, i.e. proteins that no longer have epitopes recognized by IgE, but maintain their capacity to stimulate T-cell responses [44,45]. On the other hand, stabilization of a protein can also change the B-cell epitope usage by rigidifying surface epitopes and thereby reducing the conformational space. This can lead to either loss of individual epitopes [46] or a redirection of antibody responses against specific epitopes that are not favored in the wild-type conformation [47–51]. Moreover, the presence of adjuvants in vaccine formulation has been shown to impact the protein conformation. Adsorption to alum may facilitate protein unfolding by establishing electrostatic and coordination bonds with the amino acid side chains and peptide bonds [52]. Oil-in-water emulsions have been shown to induce structural changes or even denature proteins, and also many TLR agonists (such as polymers) can interact with proteins via electrostatic interactions and possibly affect their structure [53]. These changes in protein conformation can impact on the specificity of the induced antibody repertoire [54].

There is also evidence that conformational stability might affect the route of an antigen within endosomal compartments of APCs. Hyper-stable proteins, which resist proteolysis within the antigen-processing compartment, can escape into the cytoplasm of APCs and enter the cross-presentation pathway. It has been shown that cross-presentation of APCs pulsed with soluble, virus-derived antigen can be significantly improved in the presence of chloroquine or ammonium chloride, which reduce acidification of the endosome [55]. Enhanced antigen stability may exert similar effects with regard to antigen survival during endosomal acidification and may lead to subsequent delivery of hyper-stable proteins into the cross-presentation pathway [56].

**Figure 1.**
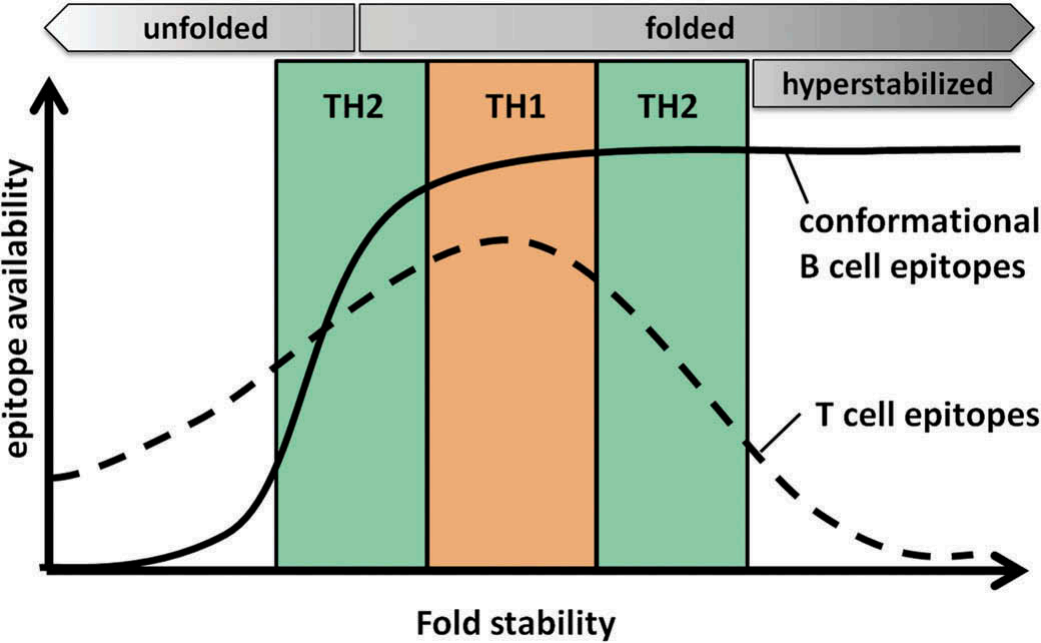
Model for the influence of protein fold stability on the availability of T and B cell epitopes. For a given protein, an optimal conformational stability is postulated, which results in efficient processing and presentation of T cell epitopes (dashed line). A high density of peptide/MHC complexes on the surface would then favor TH1 biased immune responses. Deviation from this optimum would result in too early degradation (destabilized protein), or inefficient processing in the antigen processing compartment (stabilized protein), resulting in lower peptide/MHC densities and thus TH2 polarization. Highly destabilized (unfolded) or hyperstabilized proteins display the lowest T cell immunogenicity. In highly destabilized proteins, conformational B cell epitopes (solid line) are lost due to protein unfolding, avoiding antibody responses against the folded conformation. Although B cell epitopes are maintained in hyperstabilized proteins, they induce reduced antibody responses due to lack of appropriate T cell help.

In summary, we postulate that fold stability of a protein determines the processing and presentation and thus the availability of pMHC complexes on the surface of the APC. An ‘optimal’ stability would lead to high pMHC densities, which predominantly favor TH1-polarized immune responses. Proteins deviating from this optimal stability – in either direction – provide less epitope availability and promote TH2 polarization. Protein fold stabilities at the far ends of the spectrum display very low immunogenicity, as the protein then either is in an unfolded state (highly destabilized) and thus lacks conformational B-cell epitopes or is too stable for efficient processing and presentation (hyperstabilized) and therefore cannot induce T cell help required for efficient B-cell activation (Figure 1).

### 3.1. Modulating the conformational stability of proteins

Protein molecules can be stabilized by addition of particular ions, salts, or stabilizing agents to the protein solution, via covalent modifications such as glycosylation, or by mutation of their amino acid residues. A widely used method for increasing the conformational stability of proteins is the introduction of disulfide bonds by exchange of an amino acid with a cysteine, which is far apart from another cysteine in the peptide chain, but comes spatially close in the native conformation [57,58]. As an unpaired charge inside a protein has destabilizing effects, another approach is to introduce an amino acid with an opposite charge, leading to attraction of positively and negatively charged amino acids, thereby potentially stabilizing the molecule. Also two (or more) substitutions resulting in charge pair(s) have been described to have stabilizing effects [59]. Furthermore, exchange of a small hydrophobic residue in the core of a protein with a large one (termed ‘small-to-large’ mutation) as well as ‘size-swap’ mutations matching small-to-large and large-to-small double mutations can contribute to protein stabilization. The stabilizing effect is achieved by increasing the hydrophobicity, but more importantly, by improving packing of the protein core by filling up cavities [60], termed ‘hydrophobic-core packing.’ The aforementioned methods require detailed knowledge of the protein structure and have in common that prediction of the outcome is difficult and the extent of stabilization or destabilization is unknown, until the mutated protein is expressed, purified, and characterized. Chemical crosslinking avoids this time-consuming and expensive process and has also been employed for stabilization of proteins [61,62]. Crosslinkers are chemical reagents that contain two or more reactive ends, which are capable to chemically attach to specific functional groups on proteins. Targeting of two functional groups within a single protein potentially results in stabilization of its tertiary or quaternary structure [63]. However, introduction of covalent bonds in proteins used for immunological studies focused on antigen processing might be critical because these types of bonds do not occur in nature and might impact unfolding and processing within antigen-processing compartments of APCs.

To circumvent the disadvantages of the methods described, an *in silico* mutation and screening process to generate protein variants with increased or decreased stability can be used [45,46]. Several approaches are available for this purpose. They rely on simulations employing physics-based force fields, approaches based on statistical potentials, machine learning methods, or a combination thereof. Methods utilizing physics-based force fields and/or statistical potentials, such as Eris, Rosetta, or MOE, are independent of certain stability-related training data, but in general are rather runtime-intensive. Thus, they are restricted to investigate only a comparably small number of mutations. Machine learning methods are either sequence based (e.g. MuStab, iPTREE-STAB, INPS) or structure based (e.g. INPS3D, mCSM, PoPMuSiC). They provide good prediction accuracy and in general are faster than the aforementioned methods. Hence, they are better suited for the investigation of large sets of mutations. Detailed reviews on these algorithms can be found elsewhere [49,64,65]. Another structure-based machine learning tool that we frequently use is MAESTRO, which is particularly designed for exhaustive screening of single-point as well as multi-point (de)stabilizing mutations. It predicts stability changes in terms of ΔΔG with an accuracy comparable to the leading tools in the field. The software is available either as a command-line tool [66] or as a user-friendly web interface (https://biwww.che.sbg.ac.at/maestro/web) [67]. MAESTRO utilizes various machine learning algorithms (artificial neural networks, support vector machines, and multiple linear regressions) for its predictions. The input values for the prediction include protein properties, such as its size or the environment at the mutation site, as well as statistical scoring functions, also known as knowledge-based potentials. Except for a pH value, which can be optionally set by the user, all input values are extracted from the 3D structure, which has to be provided in the form of a PDB formatted text file. The ∆∆G prediction is completed by a confidence value, which provides a sound estimation of the prediction accuracy.

MAESTRO offers four types of experiments: (1) the evaluation of user-defined mutations, (2) the calculation of a mutation sensitivity profile, (3) a scan for the most (de-)stabilizing point mutations, and (4) a scan for potential disulfide bonds. Whereas the first type is useful to investigate the effects of known mutations on the stability of a protein, the sensitivity profile as well as the scans is particularly suitable for the design of new protein variants.

**Figure 2.**
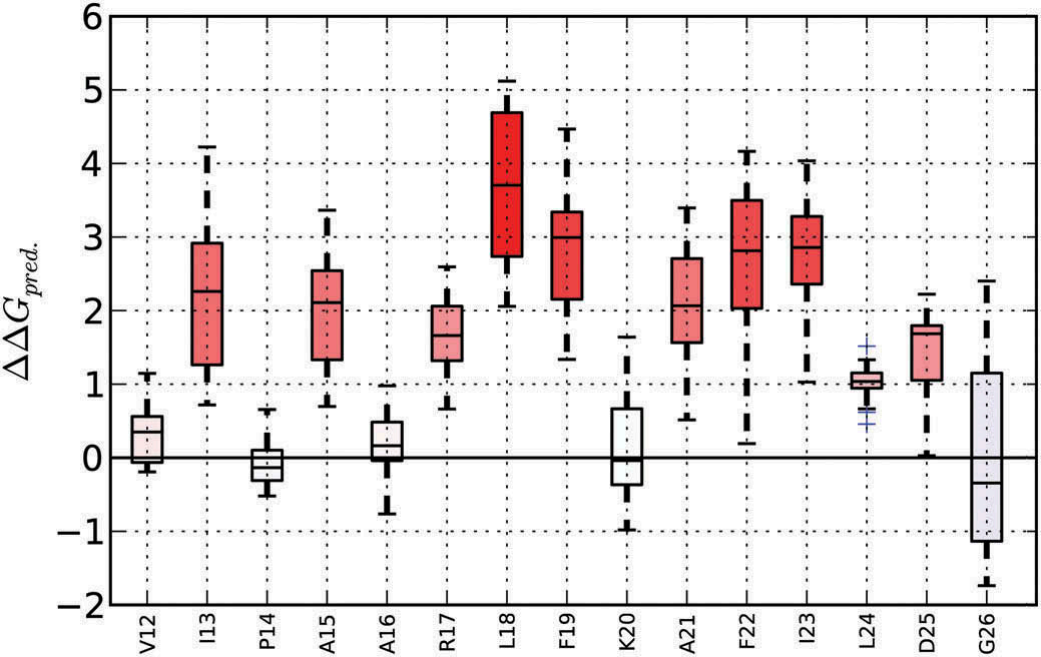
Sensitivity profile of Bet v 1.0101 (PDB-ID 4A88) created using MAESTROweb (https://biwww.che.sbg.ac.at/maestro/web). Predicted changes in Gibbs-Energy (ΔΔG_pred_ shown in kcal/mol) are presented for amino acid residues 12 to 26. At the majority of amino acid positions, an exchange to any of the other 19 possible proteogenic amino acids will result in destabilization of the protein, as indicated by an increase in ΔG (red bars). In contrast, at position 26, several possible amino acid exchanges are predicted to have a stabilizing effect (negative ΔΔG, blue bar). Full color available online.

Sensitivity profiles allow the identification of residues or protein regions, respectively, which are resistant or sensitive to mutations. Therefore, ∆∆G values are predicted for all possible point mutations in the structure and a corresponding boxplot is generated (Figure 2).

Besides the search algorithm, the user can define various constraints, like the solvent accessibility of a mutation site or a restriction to certain regions or residue types. This allows the precise design of stabilized or destabilized protein variants. With regard to immunological studies, the possibility to exclude known immunodominant T- and B-cell epitopes from the *in silico* mutagenesis helps to avoid misinterpretation of immunological changes, which are not attributable to modulation of antigen stability. Furthermore, restriction of allowed mutations to the protein core circumvents changes in or a loss of conformational B-cell epitopes. Scans result in a list of the top scoring combinations of point mutations, their predicted ∆∆G values as well as the corresponding confidence estimation.

A special scan mode for the prediction of suitable disulfide bonds is provided. Thereby, all residue pairs with a C beta–C beta distance within 5 Å are considered as potential binding partners. Then, all pairs are subsequently mutated to cysteines and evaluated. In addition to the ∆∆G value, a special disulfide bond score is computed, which takes geometric penalties into account.

The applicability on multimeric structures and on multi-point mutations as well as the provided scan modes renders MAESTRO a versatile tool for prediction of changes in fold stability [66].

### 3.2. Stability of antigens impacts immunogenicity and immune polarization

Only a few studies have been published during the last 20 years presenting data on the impact of altering the stability of protein antigens on immunogenicity, i.e. the potential to elicit cellular and/or humoral immune responses upon *in vivo* administration and/or immune polarization, meaning the capacity to promote one or the other pathway of T-cell differentiation. However, the data remain highly contradictory, with some reporting enhanced immunogenicity by increasing the conformational stability, while others found the opposite (Table 1).

**Table 1.** Effects of stabilization/destabilization on immunogenicity and immune polarization.

Antigen(s)	(De)stabilization method(s) used	Stab./Destab.	Stresses used to assess stability	Adjuvants used for imm.	Immunological outcome	Ref(s)
Snake toxin alpha	PM	+/+	pH, Tmp	-	*In vitro* T-cell proliferation ↓/↑	[68]
HEL	DSB, CL	+/+	Dgst	-	*In vitro* T-cell proliferation ↓/↑	[62]
HEL	DSB, CL	+/+	Dgst	CFA/IFA/A	IgE production ↓/↑	[69]
HEL, ML, Phl p 7	PM, CL	+/−	Tmp, Dgst	CFA/IFA/A	IgG production ↓	[70]
RIT	PM	+/−	Tmp, Dgst	-	IgG production ↓	[57]
Der p 2	PM	+/+	Dnt, Dgst	A	IgE production ↓/↑	[60]
gp120	PM	−/+	Dnt, Dgst	LT	*In vitro* T-cell proliferation ↑	[71]
RNase, HRP	Bond cleavage, cofactor removal	−/+	Dgst	A	IgG production ↓, T-cell proliferation ↓, delayed-type hypersensitivity ↓	[56,72]
Hemocyanin	CL	+/−	Dgst	CFA/IFA	Faster uptake by DCs, IgG production ↑	[61]
HIV-1 Tat	Ligand binding	+/−	Dgst	A	IgG production ↑	[73,74]
Core streptavidin	PM	−/+	Tmp	-	*In vitro* Immunoreactivity ↓	[75,76]
FMDV capsid	PM	+/−	pH, Tmp	O	Equal neutralizing antibody titers + protection	[77]
F protein of RSV	DSB, PM	+/−	Tmp, pH, Osm	Poly-ICLC	Neutralizing antibody titers ↑	[78]
F protein of RSV	CL, PM	+/−	Tmp	Poly I:C, A	Protection from viral challenge ↑	[48]
F protein of RSV	CL, DSB	+/−	Tmp, pH, Osm	Poly I:C	Neutralizing antibody titers ↑	[47]
F protein of RSV	DSB	+/−	-	LAC	Neutralizing antibody titers ↑	[50]
Bet v 1a, Phl p 5	PM	−/+	Tmp	A	IgE production ↓, IgE-binding and cross-linking ↓	[45]
Bet v 1a	PM	+/−	Tmp, Dnt, Dgst	-	IgG1 + IgE production ↑, IgG2a production ↓	[46]
RTA	PM	+/−	Tmp	A	Neutralizing antibody titers ↑	[51]

HEL: hen egg lysozyme; ML: mouse lysozyme; RIT: recombinant immunotoxin; HRP: horseradish peroxidase; FMDV: foot-and-mouth disease virus; RSV: respiratory syncytial virus; RTA: ricin toxin A chain; PM: point mutation; DSB: disulfide bond modification; CL: cross linking; Tmp: temperature; Dgst: enzymatic digestion; Dnt: chemical denaturation; Osm: osmotic stress; CFA/IFA: complete/incomplete Freund’s adjuvant; A: alum; LT: heat labile enterotoxin; O: oil adjuvant; LAC: live attenuated carrier; DC: dendritic cell.

By using differentially stable derivatives of snake toxin-α generated by introducing one or two point mutations in the core of the protein, it has been demonstrated that conformational stability of an antigen controls its proteolysis, processing, and presentation by APCs. The capacity of the variants to stimulate specific T-cell hybridomas *in vitro* inversely correlated with stability [68].

Enhanced conformational stability of hen egg lysozyme (HEL) by chemical cross-linking was also found to decrease T-cell responses *in vitro*. Whereas immunodominant epitopes were not altered by the chemical treatment and antigen uptake by APCs remained unaffected, improved resistance to proteolysis led to a decrease in generation of T-cell epitopes [62]. In a follow-up study, experiments again utilizing HEL as model antigen indicated an inverse correlation between conformational stability of the antigen and IFN-γ and IL-4 production in splenic T cells from immunized mice. The least stable variant induced the most pronounced TH2-biased response with robust IL-4 and IgE production [69]. The authors speculated that destabilized HEL leads to increased concentrations of pMHC complexes on APCs, thus accounting for the shift towards TH2 responses. This stands in clear contradiction to above cited work; however, it has been shown *in vitro* that at extremely low and extremely high concentrations of antigenic peptides, preferentially TH2 responses are induced [79].

In line with these findings, immunization with HEL derivatives and variants of mouse lysozyme induced serum IgG levels in mice, which inversely and linearly correlated with conformational stability. Furthermore, hyper-stabilization of allergen Phl p 7 almost completely prevented IgG production. The authors propose that beyond a certain threshold of conformational stability, immunogenicity of proteins would be abrogated [70].

Similar results were obtained with a stabilized variant of a recombinant immunotoxin, which retained its cytotoxic and antitumoral activity, but displayed significantly reduced antibody titers following immunization of mice [57].

By introducing point mutations into the core of the major house dust mite allergen Der p 2, one mutant with increased and one with decreased conformational stability was generated, most likely by hydrophobic core packing (increased stability) or an increase in cavity size within the hydrophobic core (decreased stability), respectively. Whereas the stabilized variant was more resistant to protease digestion, the destabilized mutant was more rapidly degraded under similar conditions. Notably, following immunization of BALB/c mice with the wild type and the mutants, animals receiving the destabilized Der p 2 variant mounted significantly elevated IgE antibody production compared to the natural allergen, whereas the stabilized mutant induced diminished IgE responses [60]. These data indicate an inverse correlation between conformational stability and allergenic potential of proteins.

CD4+ T-cell responses of individuals infected with HIV-1 are directed against a few dominant epitopes. Mucosal immunization of C57BL/6 mice with gp120 resulted in the typical pattern of dominant epitopes. However, in mice that lacked GILT, proliferative responses to the dominant epitopes of gp120 were selectively depressed, and the dominance pattern appeared to be changed. GILT-knockout (KO) mice exhibit defects in presentation of antigens that contain disulfide bonds [80]. Three gp120 variants were constructed, lacking single outer-domain disulfide bonds, and immunization of GILT-KO mice with these mutants partially restored highly proliferative responses to the dominant epitopes, whereas responses in wild type (WT) mice remained unaffected. The authors concluded that deletion of the disulfides removed the conformational barrier to presentation caused by the disulfide bonds and that efficient processing and presentation of the dominant epitopes requires local unfolding of gp120 [71].

In contrast to the aforementioned publications, another study showed that slower proteolytic degradation of internalized antigens results in longer retention of antigen in secondary lymphoid organs and enhanced presentation of T-cell epitopes [72]. As a consequence, the natural forms of RNAse and horseradish peroxidase were more potent inducers of CD4+ T-cell priming, antibody production, and delayed-type hypersensitivity reactions than their destabilized counterparts. This indicates that reduced susceptibility to degradation might not only enhance the preservation of CD4+ T-cell epitopes, but also prolong persistence of the proteins for sustained processing and presentation by DCs, and even allow for transfer of intact antigen to B cells [56,81].

A direct correlation between a protein’s conformational stability and immunogenicity was also reported by several other studies. Concholepas hemocyanin, a large glycoprotein derived from the blood of a snail, has been used as carrier for peptides and as TH1-promoting adjuvant in cancer therapy. To improve its immunogenicity, Concholepas hemocyanin was stabilized by oxidation of its carbohydrates via treatment with sodium periodate. The resulting variant was less susceptible to proteolytic digestion, and moreover, the stabilized protein was significantly faster internalized by DCs and accumulated inside endosome-like structures. Immunization of mice led to increased antibody production and an antitumor activity comparable to the wild-type protein [61].

Similarly, immunogenicity of the Tat protein of HIV-1, which represents a target for therapeutic or preventive vaccination against AIDS, was improved. Complexation with a sulfated polysaccharide rendered Tat more resistant to proteolysis, increased its immunogenicity, and simultaneously decreased its toxicity without alteration of the B-cell immunodominant region [73]. Based on these data, stabilized Tat adjuvanted with aluminum hydroxide was used for immunization of different mouse strains and cynomolgus macaques, resulting in sustained humoral and cellular immune responses [74].

Another study intended to reduce the immunogenicity of core streptavidin (for its clinical use as a drug delivery system) by inducing point mutations leading to elimination of charged or aromatic residues within B-cell epitopes. Simultaneously, residues directly involved in biotin binding and tetramerization were left untouched. This site-directed mutagenesis approach resulted in reduced thermal and chemical stability of the protein and decreased its immunoreactivity against monkey antiserum, while the functional properties of the wild-type molecule were retained [75,76].

Protein stabilization was also applied with virus capsid proteins. Empty recombinant particles have been proposed as future vaccines against picornaviruses such as foot-and-mouth disease virus. They can overcome the drawbacks of inactivated virus vaccines, i.e. expensive safety measurements during production and maintenance of a cold chain for activity. However, empty capsids are physically unstable compared to virus particles. By insertion of a point mutation leading to formation of a disulfide bridge, stabilized capsids could be produced. Immunization of cattle with the original and the mutated capsids revealed production of comparable titers of neutralizing antibodies and the same degree of protection against a viral challenge [77].

Ranked by *Science* as a runner-up for breakthrough of the year 2013, studies using structural biology approaches for rational design of human respiratory syncytial virus (RSV) vaccines have been published. Most of the first-generation RSV vaccines are based on the F-glycoprotein, a molecule which switches between pre-fusion and post-fusion states, mediating entry of RSV into cells. Synthesized as a precursor, the protein forms a trimer, which by furin cleavage turns into a mature pre-fusion F glycoprotein composed of F1 and F2 subunits. The meta-stable pre-fusion molecule spontaneously enters a stable post-fusion status, which is unfavorable as a vaccine candidate as it lacks several immunodominant epitopes. Employing structure-based design, two pre-fusion trimers have been developed, one of them stabilized by a disulfide bridge, two cavity-filling mutations, and addition of a C-terminal fibritin-trimerization domain, inducing 70–80-fold higher neutralizing antibody titers in rhesus macaques compared to post-fusion F glycoprotein [78]. Another vaccine candidate which was developed based on this rationale includes fusion of the F2 subunit to F1, two stabilizing mutations, and also an appended fibritin domain and was demonstrated to remain in the pre-fusion status for more than 50 days at 4°C. Moreover, this variant induced complete protection against viral challenge in cotton rats [48]. Based on the former molecule, four cycles of structure-based vaccine engineering were performed, resulting in second-generation F-glycoprotein vaccines with even further increased stability. This could be accomplished by an additional disulfide bond, linking of subunits, and deletion of fusion peptides, resulting in a fourfold higher neutralizing activity (in a mouse model) compared to the first-generation vaccine. The iterative process involved atomic-level design, expression and evaluation of hundreds of variants, generation of crystal structures of six mutants, and testing the immunogenicity of 14 proteins [47]. A purified recombinant RSV protein F vaccine engineered to preferentially maintain pre-fusion conformation has been recently tested in a phase I clinical study [82]. Of notice, stabilization of F protein via disulfide bond in its pre-fusion configuration expressed by a live attenuated parainfluenza virus also enhances immunogenicity [50]. Similarly, trimeric Env protein of HIV-1 has been fixed in a conformational state recognized by broadly neutralizing antibodies [49].

Ricin subunit vaccines have also been designed to elicit higher titers of neutralizing antibodies by increasing the rigidity of the immunodominant B-cell epitopes. Immunization of mice with these derivatives revealed a 5–10-fold increased efficacy to induce toxin-neutralizing IgG antibodies [51].

**Figure 3.**
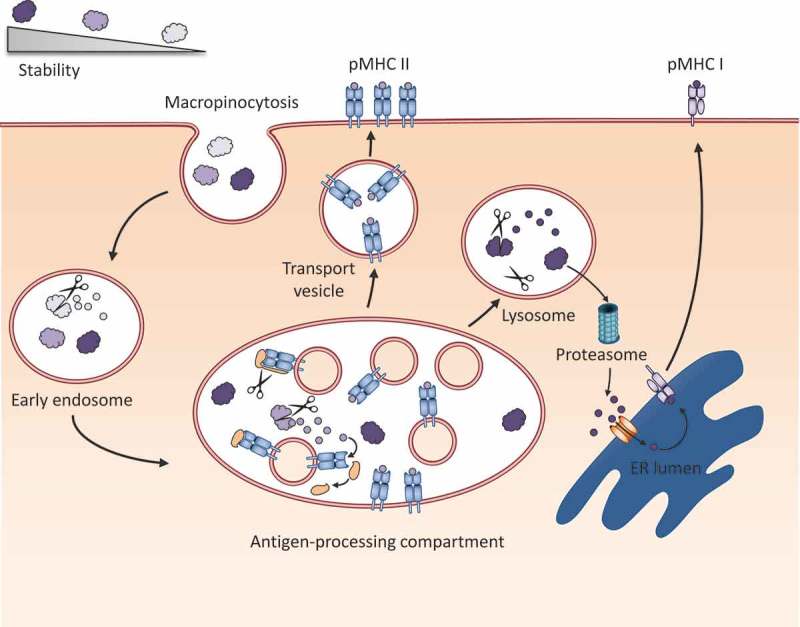
Model for the influence of protein fold stability on processing and presentation. When proteins are taken up by an antigen presenting cell, those with a very low stability can be already degraded in the early endosome, a compartment poor in MHC II molecules. Upon acidification, the early endosome matures into a late endosome, the so-called antigen-processing compartment. Proteins, which are susceptible to unfolding and processing at this acidic pH, are degraded into peptides. In the same compartment, the invariant chain, which covers the peptide binding groove of MHC class II can be cleaved, and the remaining CLIP peptide can be exchanged with a peptide of higher affinity by HLA-DM or H2-M in mice (not shown). Peptides with a very high stability can resist degradation until they finally reach the lysosome, where they are either degraded, or escape into the cytosol. Here, proteins are cleaved into peptides by the proteasome, and shuttled into the ER via TAP, where they are loaded on MHC class I (cross-presentation).

A very recent publication further contributes to the understanding of the relation between protein fold stability and immunogenicity/immune polarization. This work is based on an older publication, in which by *in silico* creation and validation, destabilized derivatives of the pollen allergens Bet v 1 and Phl p 5 with reduced IgE-binding and cross-linking capacity were generated. Upon immunization of mice, the destabilized allergens also induced decreased IgE antibody production [45]. In the recent work, an in-depth investigation of the influence of fold stability on the immunogenicity and allergenicity of the birch pollen allergen Bet v 1 was performed [46]. Excluding known T-cell epitopes from the mutation process, gradually stabilized variants of Bet v 1 were generated, harboring 1, 2, 3, or 4 point mutations. The mutant derivatives of Bet v 1 exhibited 3D structures with high similarity compared to the wild-type protein, but their thermal and chemical stability was substantially enhanced. To mimic proteolytic degradation under physiological conditions, the proteins were subjected to microsomal fractions at different pH values reflecting the acidification process during the course of endosomal maturation. The mutants as well as the wild type were poorly degradable at pH 5.9. The wild type, but none of the mutants, became susceptible to degradation at pH 5.2. At pH 4.5, the wild type was rapidly degraded, and interestingly, also the mutant harboring 4 point mutations became susceptible to proteolytic degradation. Intradermal immunization of BALB/c mice resulted in IgG titers gradually increasing with every additional stabilizing mutation. To a lesser degree, also IgE production correlated with enhanced stability. Notably, the mutant harboring four point mutations induced a strong IL-4 response, indicating a TH2-promoting capacity of this derivative. This is in line with other studies demonstrating that stabilization of the Bet v 1 molecule via ligand binding is responsible for its allergenic potential [83,84]. The data increase the complexity of the hitherto drawn picture concerning protein fold stability and its influence on immunity. The authors concluded that pH-dependent changes in conformational stability of a protein on its way through the antigen-processing compartment are decisive for induction of potent immune responses. Whereas stability at slightly acidic conditions seems to be required to resist proteolysis in the early endosome, decreased stability at lower pH and the resulting protein unfolding facilitate degradation and epitope loading on MHC II molecules in the late endosome (Figure 3) [46].

In summary, there is evidence that hyperstabilized proteins can lose their capacity to induce CD4+ T cell [46,62,68,69] and/or antibody responses [46,57,60,69,70] due to inefficient processing and presentation. Similarly, low conformational stability (ultimately resulting in unfolding) can also render the protein poorly immunogenic in terms of antibody production due to loss of conformational epitopes [45]. In contrast, the effect of a low fold stability on T-cell responses is less clear, as some proteins have been shown to be efficiently presented to T cells in an unfolded state [60,62,68,69], while others were poorly immunogenic when destabilized [56,72].

## 4. Expert commentary

Modern vaccines are often based on recombinant proteins, which can be rationally designed with excellent safety profiles and produced with standardized good manufacturing practice (GMP) procedures resulting in high purity. However, in contrast to vaccines comprising whole inactivated or killed viruses and bacteria, recombinant proteins lack inherent immunostimulatory activity and are thus often poorly immunogenic. To overcome this drawback of recombinant protein vaccines, and to specifically shape immune responses induced by immunization, vaccines are adjuvanted with immunomodulatory molecules, introduced in particulate formulations or a combination of both. Because prophylactic vaccines are frequently administered to very young individuals, safety and tolerability of adjuvants have highest priority. Moreover, for the selection of proper adjuvants, the nature of the vaccine antigens, the anticipated immune response type, the delivery method, and site of application have to be considered [1].

Significant efforts have been undertaken to develop clinically approved novel vaccine adjuvants or formulations, which meet these requirements. However, adjuvants have also been the focus of criticism, as they have been (probably falsely) implicated in the onset of autoimmune diseases [85]. In the light of results from recent publications, we are convinced that deliberate manipulation of the protein itself, i.e. antigen-inherent properties, offers enormous potential to increase vaccine efficacy and to shape the resulting immune response. Being a crucial parameter of immunogenicity and immune response polarization, optimizing the structural stability of the antigen may be an attractive alternative to high adjuvant doses with all their potential side effects and thus may result in more potent yet safer vaccines.

Based on the available data, we assume that for every given protein, there is a distinct range of stability, which may either guide the immune response towards TH1, TH2, or Treg and favor or abolish antibody responses. Such an antigen-inherent intrinsic immune bias may indeed be dominant over the immune polarizing effect of the administered adjuvant [36]. This knowledge has a large impact on vaccine design, which so far has been mainly focused on adjuvants, delivery routes, and controlled release. By combining our knowledge about the influence of the antigen structure on immunity and immune polarization with optimized adjuvants and delivery methods, it will be possible to achieve synergistic effects, which meet the desired immunological outcome of a vaccine. In addition to its potential to enhance vaccine-induced immune responses in general and to allow for tailor-made vaccine design in terms of promoting certain response types, manipulation of protein fold stability can simultaneously facilitate manufacturing, storage, and transport by rendering a continuous cooling chain dispensable [77]. Regarding all these aspects, the complexity of the immunomodulatory mechanism has to be kept in mind. Only a certain range of stability is apparently beneficial to achieve maximal immunogenicity, depending on a compromise between antigen survival *in vivo* and efficient processing and presentation by APCs. Moreover, the unfolding of a protein at a pH specific for the antigen-processing compartment plays a pivotal role for efficient induction of CD4+ T cell and antibody responses. Obviously, a ‘window of stability and pH’ defines the optimal parameters for immunogenicity of a protein [46]. This is most likely not the case for CD8+ T-cell responses, where high stability even at a low pH values can facilitate shuttling of proteins into the cross-presentation pathway. Thus, different strategies for modulating fold stability will be needed to address the specific processing and presentation requirements for a given vaccine.

Summing up, fine-tuning of pH-dependent fold stability enables the rational design of protein derivatives with desired properties, ranging from highly immunogenic antigens for vaccine use to hyperstabilized and non-immunogenic proteins as required for biologicals.

## 5. Five-year view

We are currently just beginning to unravel the intrinsic effects of fold stability on the immunogenicity and immune polarization and its implementation in the vaccine field. A major limitation today is that the rational design of stabilized and destabilized proteins is still an error-prone process. Moreover, the term ‘stability’ itself is not unambiguous as it encompasses thermal, chemical, or pH stability. Especially, the latter was not addressed in most of the available studies, yet turned out to be a key factor, crucial for the proper processing/presentation of antigenic peptides. A broader success of this approach in the vaccine field will depend on the development of high-throughput methods for evaluating antigen-processing and pMHC density in physiological settings. However, first examples of stability optimized vaccines have been demonstrated, and we expect further breakthroughs with individual vaccine molecules within the next years.

We also are convinced that the recently gathered knowledge will trigger more studies with the focus on understanding how pMHC/TCR avidity modulates immune responses and how innovative next-generation vaccines can be designed that meet the needs of patients.

## Key issues

- Antigen processing and presentation is crucial for immunogenicity of antigens, i.e. the ability to induce T cell responses and/or production of antibodies.
- Differentiation of T cells into distinct effector populations is controlled by the cytokine milieu and the strength of the TCR signalling.
- Conformational stability of protein antigens determines their fate within the antigen processing compartment of antigen presenting cells.
- Stabilization/Destabilization of antigens has been demonstrated to influence immunogenicity and immune polarization.
- For any given protein antigen there is a degree of stability, which leads to induction of TH1, TH2, or Treg responses and also determines antibody production.
- Unfolding of a protein at a pH specific for the antigen processing compartment plays a pivotal role for efficient induction of CD4 + T cell and antibody responses
- By using software for prediction of changes in stability upon point mutations, vaccine candidates with the desired stability profile can be selected for expression and characterization.
- Manipulation of the antigen itself has the potential to revolutionize future development of adjuvant-free vaccines.
